# A High-Grade Glioma of Temporal Lobe in a Child: A Case Report and Literature Review

**DOI:** 10.7759/cureus.11802

**Published:** 2020-11-30

**Authors:** Aadil M Khan, Manal Dawe, Warda Shahnawaz, Muhammad W Saleem, Zahoor Ahmed

**Affiliations:** 1 Medicine, Ganesh Shankar Vidyarthi Memorial Medical College, Kanpur, IND; 2 Medicine, Capital Medical University, Beijing, CHN; 3 Medicine, Jinnah Sindh Medical University, Karachi, PAK; 4 Internal Medicine, Hayatabad Medical Complex Peshawar, Peshawar, PAK; 5 Internal Medicine, King Edward Medical University, Mayo Hospital, Lahore, PAK

**Keywords:** high-grade glioma, malignant glioma, radiotherapy, chemotherapy

## Abstract

High-grade glioma is also called a malignant glioma because it is fast-growing and spread rapidly through brain tissue. Due to the rarity of high-grade glioma, its diagnosis and management are multi-faceted. We present a case of a 10-year-old girl presented with headache, seizure, and right-sided weakness of upper and lower limbs. Neurological exam revealed reduced power in both upper and lower right limbs with reduced sensation and reflexes. Magnetic resonance imaging revealed an ill-defined altered signal intensity mass involving the left temporal lobe with parenchymal involvement and surrounding perilesional vasogenic edema. Biopsy of the lesion confirmed high-grade glioma. The patient underwent external beam radiation therapy with concomitant daily temozolomide treatment, followed by adjuvant standard temozolomide. However, progressive neurological worsening and an increased lesion size led to partial tumor resection through a craniotomy to remove intracranial hypertension, which was unsuccessful, and the patient could not survive after the procedure.

## Introduction

High-grade gliomas are the neoplasms of the glial cells found in the central nervous system. These neoplasms are fast-growing and spread rapidly through the brain tissue. These tumors are uncommon, especially in children [1]. High-grade gliomas can be classified based on their location and their appearance under a microscope. Due to the rarity of high-grade gliomas, their diagnosis and management are complex and controversial, and the extent of the lesion is predominately related to patient survival [2]. Herein we present a case of high-grade glioma in a girl presented with seizure.

## Case presentation

A 10-year-old girl with a family history of colon cancer presented to the emergency department with generalized tonic-clonic seizure. She also complained of a similar episode two weeks ago. Her past medical history was associated with a prolonged morning headache, which was generalized, dull, non-radiating associated with nausea for the last two months. She reported difficulty in walking and weakness in the right upper and lower extremity associated with reduced sensation in both upper and lower limbs. She had no history of trauma or fall.

Initial evaluation revealed a temperature of 98°F, blood pressure of 120/70 mmHg, heart rate of 95 beats per minute, respiratory rate of 20/minute, and oxygen saturation of 99% on room air. On physical examination, she was confused with incoherent speech. On neurological examination, her power was 2/5 in both upper and lower right limbs, and coordination was intact with reduced sensory sensation. Her toes were down going, and the reflexes were also reduced on the right side of the body. Her cranial nerve examination was not significant, with no evident deformity on her face.

**Figure 1:**
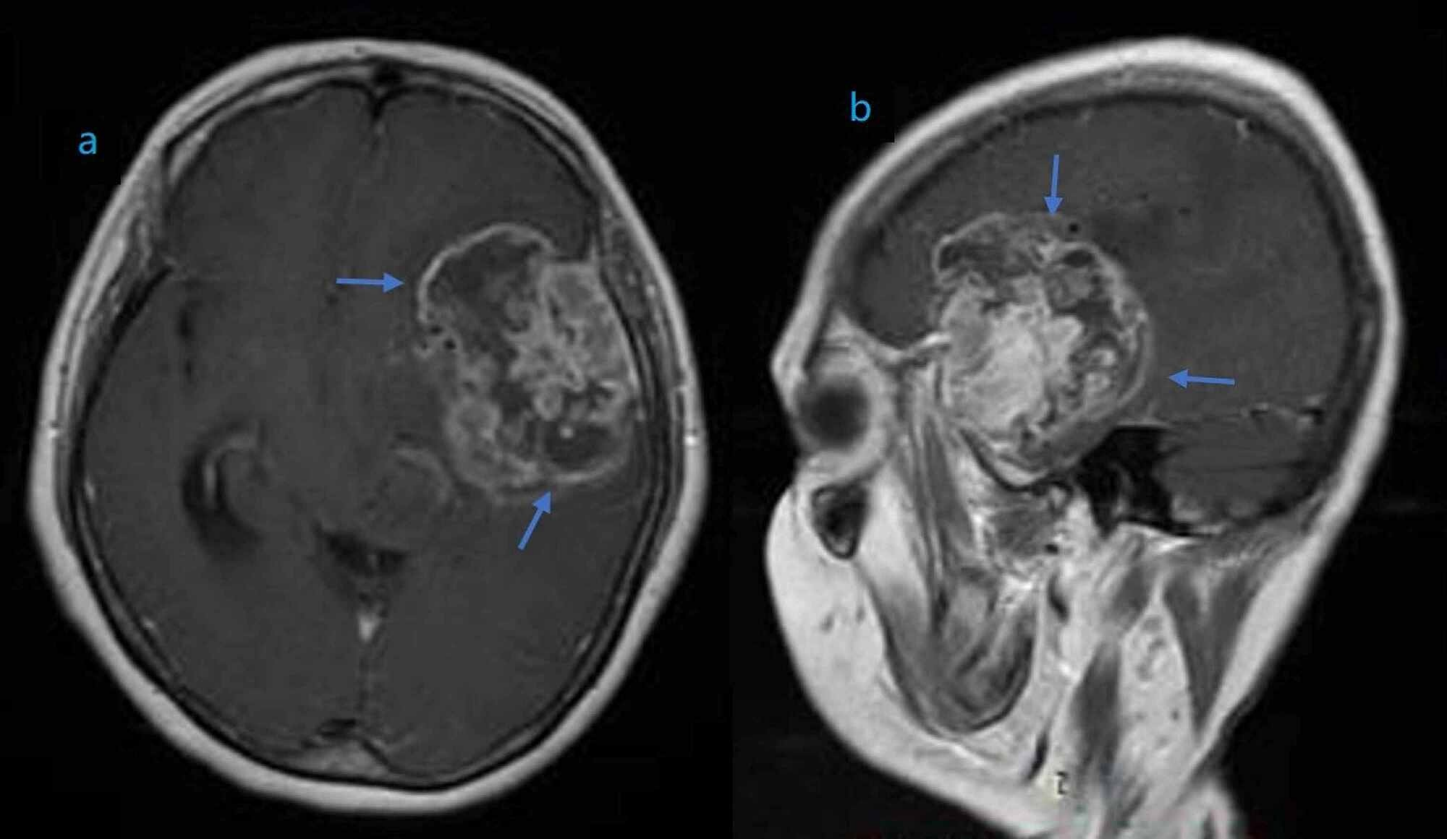
Magnetic resonance imaging (MRI) of the brain showing an enhancing mass of heterogeneous density involving the left temporal lobe in coronal (a) and sagittal (b) planes.

**Figure 2:**
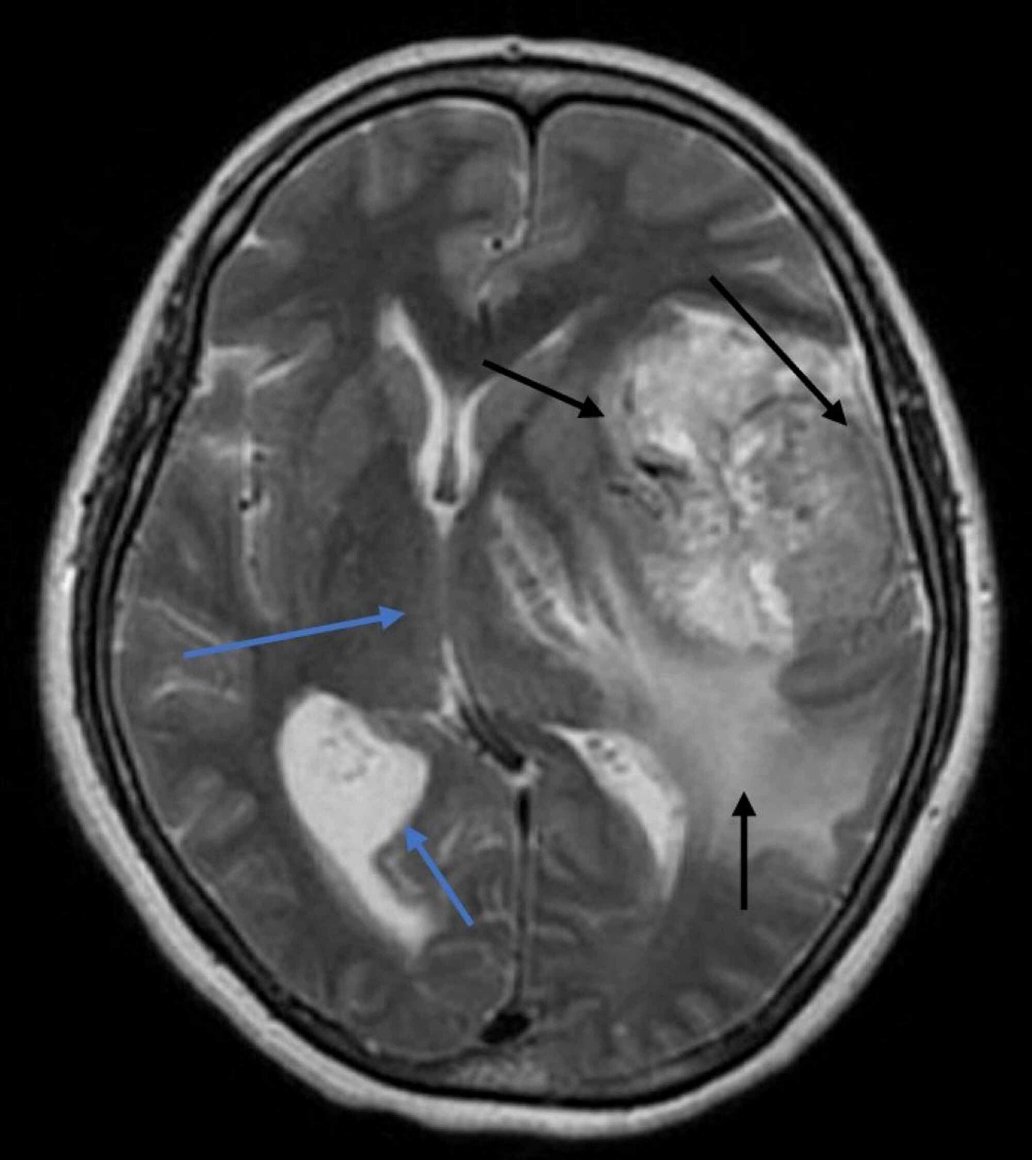
Axial FLAIR T2-weighted image of the patient’s hemisphere, showing hypointense to hyperintense irregular mass with parenchymal involvement and perilesional vasogenic edema (black arrows). The mass was causing midline shift, and mild dilation of the contralateral lateral ventricle was also identified (blue arrows). FLAIR: Fluid-attenuated inversion recovery.

Her signs and symptoms were suggestive of the central lesion, and magnetic resonance imaging (MRI) of the brain was performed, which revealed an ill-defined altered signal intensity mass involving the left temporal lobe, retrieving hypointense signal on T1-weighted image (T1W1), isointense to hyperintense on T2/FLAIR (fluid-attenuated inversion recovery) (Figure 1). Parenchymal involvement was also noted with surrounding perilesional vasogenic edema. The mass was causing midline shift, and mild dilation of the contralateral lateral ventricle was also identified (Figure 2). MRI of the spine was normal.

The patient underwent the biopsy, which revealed high-grade glioma, which demonstrated the cells with high mitotic activity as well as hyperchromatic and pleomorphic nuclei. On immunohistochemical examination, the tumor was positive for glial fibrillary acidic protein (GFAP), Ki-67 cellular proliferation antigen, and S-100 protein. The patient underwent external beam radiation therapy with daily temozolomide, followed by adjuvant standard temozolomide, for five consecutive days, every 28-day cycle. However, her condition worsened over the month with hemiplegia and a fluctuating level of consciousness. An increased lesion size led to partial tumor resection through a craniotomy to remove intracranial hypertension, which was unsuccessful, and the patient could not survive after the procedure.

## Discussion

High-grade gliomas are the most challenging tumors in terms of treatment. Cerebral tumors are the most common solid neoplastic tumors in childhood and are the prime cancer-related cause of death [3]. Of all brain tumors, glioma communally accounted for 27.5% and 80% of the malignant brain tumors [3]. Different parts of the brain are the more frequent sites for high-grade gliomas than others. The top three most common sites for high-grade gliomas in the central nervous system involve the frontal lobe (25.9%), the temporal lobe (19.8%), and other brain areas (19.4%) [4]. The rarest sites for malignant gliomas are the pineal (0.1%), the meninges (0.1%), and the cranial nerves (1.2%). Most of the high-grade gliomas occur within the four cerebral lobes (60.8%), which also signify the majority of the malignant brain tumor sites (54.1%). Among all the cerebral tumors, gliomas constitute approximately 60%, and half of them are included in high-grade gliomas. The prognosis of high-grade glioma is poor, and five-year survival rates vary from 5% to 15% [5]. The five-year survival rates by cortical location differ: frontal lobe (34.3%), temporal (23.0%), parietal (19.6%), and occipital (20.8%). The malignant tumors of the parietal lobe have been the lowest 10-year survival rate of 14.3% [4].

**Figure 3:**
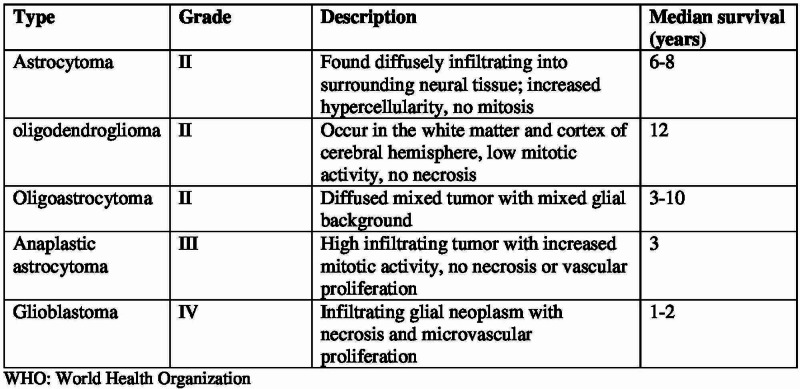
WHO 2007 classification for diffuse gliomas

Gliomas are graded on cytogenic features and degree of malignancy after hematoxylin and eosin staining. Currently, gliomas are graded via the World Health Organization (WHO) grading scale (Figure 3) [4]. This scale was first employed in 1920 when Bailey and Cushing first classified the glial tumors by their resemblance to known glial cell types: astrocytes, oligodendrocytes, etc. Infiltrating gliomas are graded as WHO grade II-IV, and grade I tumors are classically solid and non-infiltrative such as pilocytic astrocytomas. Histologic grading is based on findings of nuclear atypia, mitotic activity, necrosis, and microvascular proliferation. In our case, the patient had grade IV glioma with predominant neurological features [4]. Both grade II and grade III gliomas have a pronounced tendency to recur or progress to grade IV status and may merely represent precursor stages to grade IV [4].

The clinical presentation of high-grade glioma chiefly depends on the patient’s age and the tumor localization. These neoplasms present with different signs and symptoms, and neurological impairment is predominately quick, varying from months to days. A seizure can be the presenting symptom and may herald the onset, particularly when neoplasms are adjacent to the cerebral cortex. A headache, visual deficit, and hemiparesis are the other clinical manifestations in gliomas. In our case, the first clinical manifestation was seizure with chronic headache and signs of hemiparesis [6]. The diagnosis of high-grade glioma is based on the clinical presentation, imaging modalities, and biopsy. A brain MRI is an investigational tool of choice for gliomas [6]. However, for a definitive diagnosis, a brain biopsy is required. The treatment of high-grade glioma is still a challenge. Surgery is the primary treatment for high-grade gliomas if it can be done safely. Chemotherapy and radiotherapy also have satisfactory results, but these are associated with high morbidity [7,8]. Current guidelines for glioma treatment include tumor resection, chemotherapy, and local radiotherapy.

Symptomatic management of high-grade gliomas is mandatory. Seizures and cerebral edema can cause adverse neurologic symptoms that are life-threatening. Antiseizure medications are used to control seizures caused by brain tumors. Cerebral edema is managed with glucocorticoids, most commonly dexamethasone. The dexamethasone dose is decreased gradually to the lowest level to minimize the side effects by controlling symptoms. Hydrocephalus is uncommon in gliomas. However, treatment may be required in the form of surgery to place a shunt. Deep vein thrombosis is usually managed with anticoagulant medicines [9].

The use of adjuvant chemotherapy for the treatment of high-grade gliomas was reputable in the 1980s. In a study by Sposto et al., it was reported that lomustine and vincristine showed significant results against high-grade glioma [10]. Similarly, lomustine and temozolomide have proved to be effective in increasing survival rates [11]. Temozolomide is usually taken orally daily with radiation and then for up to six monthly cycles (five consecutive days every four weeks) after the radiation. Chemotherapy is only effective in newly diagnosed high-grade gliomas. Oral lomustine is an option in this situation. These studies have demonstrated that chemotherapy, radiotherapy, and surgical resection of the tumor were unsuccessful in accomplishing the long-term survival rates. Further research is warranted so that better treatment options may be developed to enhance the quality of life and disease prognosis. 

## Conclusions

High-grade gliomas are rare tumors associated with an unfavorable prognosis, despite the development of treatment options. Our patient developed intracranial hypertension, and we chose to perform both chemotherapy and radiotherapy. The unfavorable tumor progression prohibited the use of chemotherapy and led us to partial tumor resection, which was eventually unsuccessful. Poor prognosis despite the evolution of treatment warrants further research to improve the quality of life and better disease management.
